# Synchronization Stability Model of Complex Brain Networks: An EEG Study

**DOI:** 10.3389/fpsyt.2020.571068

**Published:** 2020-12-04

**Authors:** Guimei Yin, Haifang Li, Shuping Tan, Rong Yao, Xiaohong Cui, Lun Zhao

**Affiliations:** ^1^College of Information and Computer, Taiyuan University of Technology, Taiyuan, China; ^2^Department of Computer Science, Taiyuan Normal University, Jinzhong, China; ^3^Center for Psychiatric Research, Beijing Huilongguan Hospital, Beijing, China; ^4^School of Educational Sciences, Liaocheng University, Liaocheng, China

**Keywords:** complex brain networks, a synchronous stability model, EEG, random apollonian networks, block coordinate descent

## Abstract

In this paper, from the perspective of complex network dynamics we investigated the formation of the synchronization state of the brain networks. Based on the Lyapunov stability theory of complex networks, a synchronous steady-state model suitable for application to complex dynamic brain networks was proposed. The synchronization stability problem of brain network state equation was transformed into a convex optimization problem with Block Coordinate Descent (BCD) method. By using Random Apollo Network (RAN) method as a node selection rule, the brain network constructs its subnet work dynamically. We also analyzes the change of the synchronous stable state of the subnet work constructed by this method with the increase of the size of the network. Simulation EEG data from alcohol addicts patients and Real experiment EEG data from schizophrenia patients were used to verify the robustness and validity of the proposed model. Differences in the synchronization characteristics of the brain networks between normal and alcoholic patients were analyzed, so as differences between normal and schizophrenia patients. The experimental results indicated that the establishment of a synchronous steady state model in this paper could be used to verify the synchronization of complex dynamic brain networks and potentially be of great value in the further study of the pathogenic mechanisms of mental illness.

## Introduction

Synchronization of complex networks is a very important research direction in the study of complex network dynamics because the complexity of the structural characteristics of complex networks has a multifaceted impact on the dynamics of synchronization. This direction has been a focus of research in recent years (1–5). At present, most of the synchronization research on complex networks has been done to investigate the impact of topology on the synchronization capability, but the synchronization process *per se* has rarely been studied. In fact, the synchronization process is very important, because synchronization is a gradual process. Research on the synchronization process is helpful for revealing the evolutionary mechanism of complex systems and for exploring interesting phenomena that occur when a network reaches global synchronization. In 1990, Pecora and Carroll pioneered the study of chaotic synchronization (6). Since chaos synchronization potentially has important applications in many fields, many scholars have carried out research into chaos control and synchronization, and researchers have proposed a series of effective methods for realizing chaotic synchronization. Interestingly, the study of complex networks has boomed since the publication of two ground breaking articles, “Small World Networks” in Nature in 1998 (7) and “Scale-free Networks” in Science in 1999 (8). The synchronization of complex networks has been also widely studied. Chen et al. studied synchronization and stability models of complex networks with coupled oscillator-based continuous systems (8, 9). Their models primarily explored the influence of network structures on the synchronization stability of dynamic networks, and their work focused on synchronization theories that corresponded to different types of complex networks.

However, investigating how to determine each parameter for a specific application area could provide more practical applications. Yu et al. (10) constructed a discrete modular neuronal network made of small-world sub-networks based on a map-based neuron model [proposed by Rulkov (11)] to investigate its synchronization mechanisms. Although their model reflected that the variations in coupling strengths and the probability of random links between different sub-networks can induce synchronization transitions, it was a discrete neuronal network. In reality, neuronal networks are more likely to be sequential complex networks. Arenas et al. (2) revealed the structural characteristics of this type of network by analyzing the synchronization process of the network. However, the method is only applicable to a community network with a hierarchical structure. In fact, many community networks do not have a hierarchical structure. So this method needs to be further improved to increase its applicability. To better understand the dynamic behavior of complex brain networks, the nonlinear method can be used to analyze the synchronization of a chaos system. The main application of this method is the phase synchronization method, which divides the amplitude from the phase information and considers only the phase information so that the stability of the signal is not as much of an issue. To this end, Zhou et al. (12–14) put forward a segmentation prony method, which improved the frequency and phase resolutions, thereby enhancing the anti-noise ability of simultaneous judgments. However, the synchronicity of the brain in different frequency bands is ubiquitous in brain integration and needs further research. Therefore, the characteristics of the synchronous oscillation of the brain network were studied from the perspective of complex networks in this paper. Based on the theory of synchronization stability in modern cybernetics as well as on the global features and local features of the brain network (15–18), a synchronization steady state model suitable for the brain network was established and a theoretical basis for judging whether the brain network reaches a synchronization state was also developed. According to the theory of complex networks, the synchronization mechanism was further explored in brain networks from different frequency bands of signals, which is of great significance to the development of brain science. The rest of this paper is organized as follows.

PRELIMINARIES
  Definition of complex network synchronization
  Synchronization criteria of complex network -master stability function (MSF)
  C. Sub network nodes selection rules
  Complex Dynamic Brain Network Synchronization (CDBNS) Model
    CDBNS model and proof
      CDBNS model
      Proof and Optimization of CDBNSs
    Synchronization stability discriminant construction of CDBNS model
  Numerical Experiment
    Experimental simulation and result analysis on alcoholism addiction data set
      Simulation results analysis of alcoholism addiction data set
      Difference analysis of synchronization ability
    Experimental simulation and results analysis on working memory data of schizophrenia
      Simulation results analysis of working memory data of schizophrenia
      Difference analysis of synchronization ability
  Discussion

The basic concepts of complex network synchronization used in this paper are introduced in section 1. Section 2 establishes a complex dynamic brain network synchronization model (CDBNS), along with the proof and optimization of this model. Section 3 provides experimental results and a discussion of the results and Section 4 includes the conclusions and suggestions for future work.

## Preliminaries

A general complex dynamical network synchronization model and several mathematical preliminaries are introduced in this section.

### Definition of Complex Network Synchronization

Network synchronization is a very common and very important non-linear phenomenon. There are many different research methods of study network synchronization (17), such as common constant synchronization, phase synchronization, generalized synchronization, etc. Identical synchronization is defined as (18, 19):

**Definition1**: Let x*i* (*t*, X_0_) be a solution of the complex dynamic network

(1) $$\begin{array}{l} ẋ=f\left(x_{i}\right)+g_{i}\left(x_{1},x_{2},x_{3},\ldots x_{N}\right),\;i=1,2,\ldots ,N \end{array}$$

where, $X_{0}={\left({\left(x_{1}^{0}\right)}^{T},{\left(x_{2}^{0}\right)}^{T},\ldots ,{\left(x_{N}^{0}\right)}^{T},\right)}^{T}\in R^{N*N},f:D\to R^{n}$ *and* $g_{i}:D\times D\to R^{n}\left(\;i=1,2,\ldots ,N\right)$ are all continuously differentiable, *D* ⊆ *R*^*N*^ and meet *g*(*x*_1_, *x*_2_, …, *x*_*n*_) = 0. There is any non-empty open set *C* ⊆ *F* in the domain, which can make any *x*_*i*_ (*t, X*_0_) ∈ *F* and lim||*x*_*i*_(*t, X*_0_) − *s*_*i*_(*t, X*_0_)|| = 0 *i* = 1, 2, …, *N* for any $x_{i}^{0}\in C,i=1,2,\ldots ,N\;$and *t* ≥ 0, *i* = 1, 2, …, *N*, where *s* (*t, X*_0_) is an effective solution space of equation $x_{i}^{0}\in C,i=1,2,\ldots ,N$ and then complex dynamics network (1) can reach an identity synchronous steady state, and C × *C* × · · · × C is called the synchronous area of the complex dynamic network (1).

Identical synchronization, which means that all nodes in the network are in the same state at some time point, is a common phenomenon in network synchronization. In Definition 1, *s* (*t, X*_0_) is the synchronous steady state of the network, and *x*_1_ = *x*_2_ = … = *x*_*N*_ is the synchronization manifold of network state space; that is, each physical oscillator tends to be in a described state when the network is in synchronization.

### Synchronization Criteria of Complex Network -Master Stability Function (MSF)

Three kinds of criteria control complex network synchronization. One is the Lyapunov function, which is used to analyze global stability; the second is master stability function (MSF) proposed by Pecora and Carroll in the 1990s (20), which is primarily used to analyze the local stability; and the third is connection graphic stability method established in 2004 by Belykh et al. (21, 22), which is used to analyze the global synchronization of the time-varying network, and the third method combines Lyapunov function method and graph theory. In this way, this method can avoid computing the Lyapunov exponent and the eigenvalue of the Laplacian matrix. The second method (MSF) is used in the model proposed in this paper. The definition of the master stability function (MSF) (20) is as follows:

**Definition 2:** Consider a continuous time dissipative coupling dynamic network

(2) $$\begin{array}{l} \dot{x_{i}}=f\left(x_{i}\right)-c\overset{N}{\underset{j=1}{\sum }}l_{ij}H\left(x_{j\;}\right),\;i=1,2,\ldots ,N \end{array}$$

where $\dot{x_{i}}=f\left(x_{i}\right)\;$is the dynamic function of node i, $x_{i}={\left(x_{i1},x_{i2},\ldots ,x_{in}\right)}^{T}\in R^{N}$is the state variable of node *i*, constant *c* > 0 is coupling strength of network, *H* ∈ *R*^*N*×*N*^ is the internal coupling function between the state variables of each node, *L* = (*l*_*ij*_)_*N*×*N*_ is Laplacian matrix which is defined as: if there is a connection from node *i* to node *j*, then *l*_*ij*_ = 1, otherwise, *l*_*ij*_ = 0 and *L* satisfies the dissipative coupling condition *l*_*ij*_ = 0. Suppose that *s*(*t*) is a periodic solution of system $\dot{x_{i}}=f\left(x_{i}\right)$, we linearize network (2) at *X*(*t*) = *S*(*t*), and now let

(3) $$\begin{array}{l} \eta_{i}\left(t\right)=x_{i}\left(t\right)-s\left(t\right),\;i=1,2,\ldots ,N \end{array}$$

and substitute (3) into network (2) to get

(4) $$\begin{array}{l} {\dot{\eta }}_{i}=Df\left(s\right)\eta_{i}-c\overset{N}{\underset{j=1}{\sum }}l_{ij}DH\left(s\right)\eta_{j}i=1,2,\ldots ,N \end{array}$$

where *Df* (*s*) is the value of Jacobian *f* (*x*) at *x* = *s*(*t*). Let $L^{T}=Pdiag\left(\lambda_{1},\lambda_{2},\ldots \lambda_{n}\right)P^{-1}$ and $\hat{\eta }=\left[{\hat{\eta }}_{1},{\hat{\eta }}_{2},\ldots ,{\hat{\eta }}_{N}\right]=\left[\eta_{1},\eta_{2},\ldots \eta_{N}\right]P$, λ_*i*_ is the eigenvalue of external coupling matrix L, then system (4) can be rewritten as

(5) $$\begin{array}{l} {\dot{\hat{\eta }}}_{i}=\left[Df\left(s\right)-c\lambda_{i}DH\left(s\right)\right]{\hat{\eta }}_{i},\;i=2,3,\ldots ,N \end{array}$$

A common criterion to judge the synchronous stability of a system is to require that the Lyapunov exponents for (2) are all negative (1, 18). But this does not mean that this condition alone can determine whether a system realizes a steady state or not. In (5), only $\hat{\eta }\;$and λ_*i*_ are related to *i*. Considering that the eigenvalues of external coupling matrix *L* can possibly be complex numbers, the main stability equation of network (4) is defined as:

(6) $$\begin{array}{l} \dot{\hat{\eta }}=\left[Df\left(s\right)-c\left(\alpha +i\beta \right)A\right]{\hat{\eta }}_{i} \end{array}$$

Maximum Lyapunov exponent of (6) is the function of two real variables α and β, and is the master stability function of complex dynamic network. For a fixed network coupling strength and with a value of i obtained by iterative calculation, this equation can determine the unique point c in the complex plane that is determined by the two real variables α and β. The positive and negative values of *LE*_max_ corresponding to this point reflect the stability of the characteristics of the model. If starting from a value >i, all of the characteristics of this model are stable, then the entire network of the strength *c* is called an asymptotically stable network. That is, when the transversal Lyapunov exponent is negative and the *LE*_max_ is positive, then we can judge whether a network is asymptotically stable. When the network is a simple graph with no exponential terms and no direction, the MSF is ẏ = [*Df*(*s*) − *cαA*]*y*. Because brain networks based on EEG signals are complex networks, we cannot directly eliminate the plural items; the specific form will be proved in the next section.

### Sub Network Nodes Selection Rules

**Figure 1:**
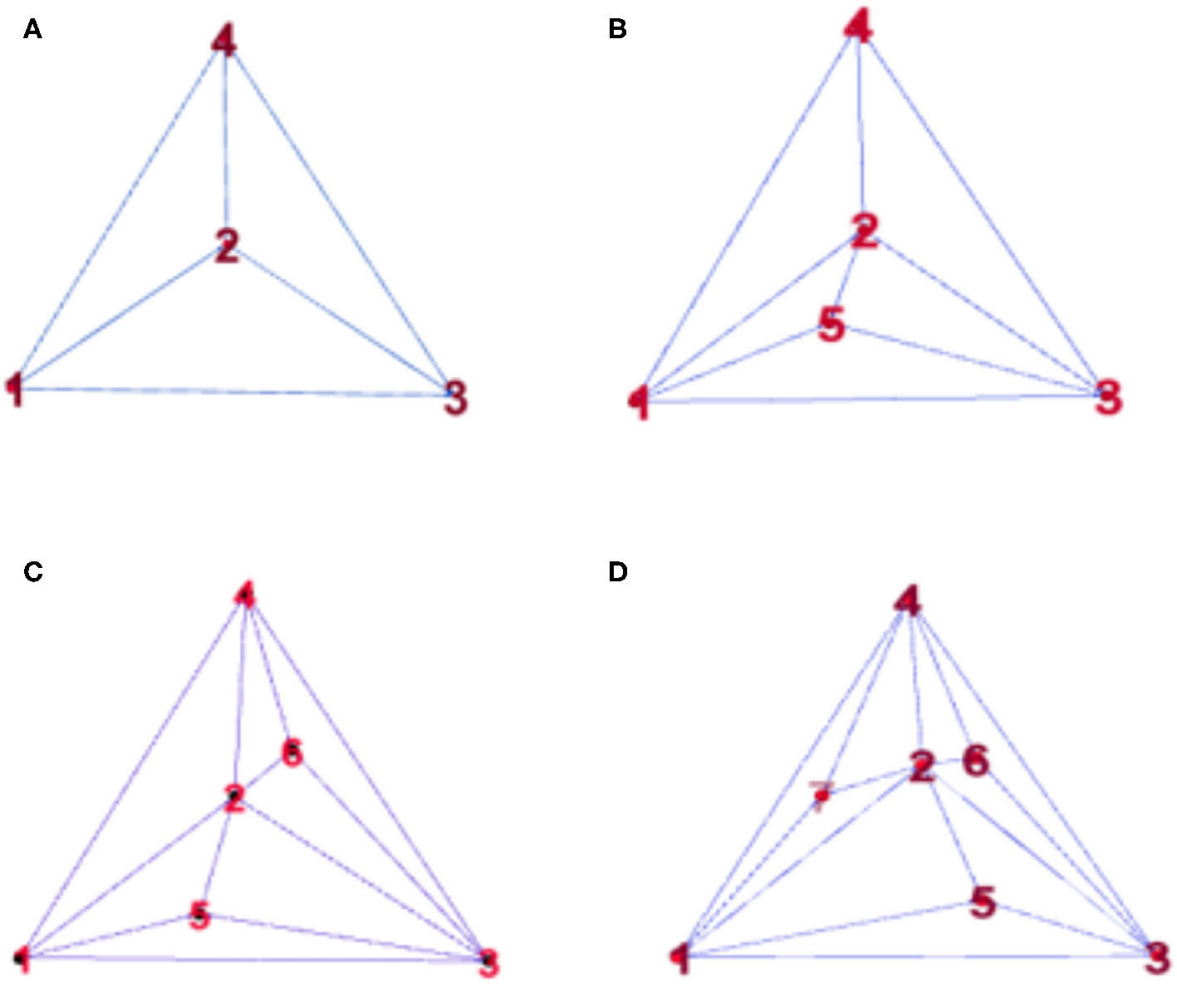
Snapshots of a random Apollonian network (RAN) at: **(A)** *t* = 1, **(B)** *t* = 2, **(C)** *t* = 3, **(D)** *t* = 4.

To observe the synchronization changes dynamically during the experiment, the method in this paper used Random Apollonian networks (RANs) (23, 24) to add nodes to generate dynamic networks because the networks generated using this rule have high clustering coefficients and smaller average distances. The maximum of the minimum nonzero eigenvalue of the Laplacian matrix is relatively large; that is, the generated networks have the small-world and the scale-free attributes of the random Apollo network, and are closer to the actual complex network than a small-world network or a scale-free network. Random Apollonian network (25, 26) is a popular random graph model for generating planar graphs with power law properties. We briefly describe the model as follows. A random Apollonian Network starts with a triangle containing three nodes marked as 1, 2, and 3. Then, at each time step, a triangle is randomly selected, and a new node is added inside the triangle and linked to the three vertices of this triangle. The sketch maps for the network growing process are shown in Figure 1. It is clear that, at time step t, our network is of the order *N* = *t* + 3. Using this simple rule, the person making the model can get a random Apollonian network of any arbitrary order that he likes. At time step 1, the fourth node is added to the network and linked to nodes 1, 2, and 3. Then, at time step 2, the triangle Δ123 is selected, and the fifth node is added inside this triangle and linked to nodes 1, 2, and 3. After that, the triangles Δ234 and Δ124 are selected at time steps 3 and 4, respectively. Nodes 6 and 7 are added inside these two triangles respectively. Figure 1D shows a random Apollonian network of order 7. Continuing with similar iterations, the modeler can get RANs of any order that he chooses. Note that the selected three nodes must connect each other during the generation of RANs, and the modeler must ensure that each triangle can only be selected once in the process of generating the whole network. In the process of updating the local sub-network, the block coordinate descent method is used to optimize the generated subnet. The optimization process is as follows: firstly, the non-negative matrix of the brain network nodes is decomposed, and then the dynamic network is processed by extermination. In this way, the influence of the change of the maximum between ness and average distance on the synchronization stability of the network can be avoid, which can better help us to judge the brain network synchronization stabilization.

## Complex Dynamic Brain Network Synchronization (CDBNS) Model

It is a non-convex and non-smooth problem to judge the synchronization stability of brain network. UV - decomposition theory (27) is an important method to study non-smooth convex optimization. In this section, we propose a complex dynamic brain network synchronization model, and transform the stability problem of judging the equation of state of brain network into convex optimization problem by using UV decomposition theory.

### CDBNS Model and Proof

In this section, a complex dynamical brain network model is established based on the general complex dynamical network model introduced in section Definition of Complex Network Synchronization.

#### CDBNS Model

CDBNS Model to study the dynamic synchronization of complex brain network, a dynamic system in each node of the network is usually defined. The dynamic system can be either a limit cycle or a chaos cycle. Also, a mutual coupling effect occurs between the dynamic system of the two connected nodes so it is termed a dynamic network. Strictly speaking, the brain network has a total of N edges; the N edges are abstracted into N nodes in the network, and the nodes in the same brain area constitute the connected edges, that is, a unified network system. Based on the relationship between the brain network and the complex network, the node state variables are generated by randomly selecting the nodes in the network (generation method see section Sub Network Nodes Selection Rules). Based on Lyapunov stability in the complex network, a CDBNS model is proposed. This model describes the synchronization feature of the brain network and examines whether the local brain nodes have similar synchronizations. Let *W* be the set of total brain nodes and *M, G* be the factorization initial reference matrix of the random selected nodes. We use a significance test to verify whether the assumption is true. If it is true, then we can perform matrix factorization $S_{i}^{T}=U_{i}\Lambda U_{i}^{-1}$ where Λ = *diag*(λ_1_, λ_2_, …, λ_*n*_) and $V_{i}=U_{i}^{-1}$. Then the norm ${\left\|S_{i}-U_{i}V_{i}\right\|}_{F}^{2}\;$ *F* holds the oscillation information of the valuable nodes in the brain network. Substituting it into (2), a general complex network dynamic model composed of n identical and diffusive coupled nodes is established. The state equation of the brain network is described as

(7) $$\begin{array}{l} \dot{x_{i}}=f\left(x_{i}\right)-\overset{n}{\underset{j=1}{\sum }}cl_{ij}\;\underset{\left\{W_{i},M_{i},G_{i}\right\}}{min}\overset{m}{\underset{i=1}{\sum }}\frac{1}{2t}{\left\|S_{i}-U_{i}V_{i}\right\|}_{F}^{2} \end{array}$$

where *c* is the coupling strength of the brain network that we constructed, all the *xi* = (*x*_*i*__1_; *x*_*i*__2_; · · ·*; x*_*in*_)^*T*^ ∈ *R*^*N*^ are the state variables of the nodes, *t* is length of the EEG signal sampling time, *L* is the Jacobian matrix of this network, all the *l*_*ij*_ are the elements of matrix *L*, each *V*_*i*_ is the column vector for each *U*, and *f* (*x*_*i*_) is the EEG dynamic equations proposed by Liley (28), which uses a method of mathematical analysis based on the physiological structure and anatomical basis of the cerebral cortex neuron network. There are 60 nodes in S (In the experiment, a 64-conductor EEG acquisition device was used, horizontal and vertical eye electrodes are excluded). In accordance with the rules introduced in section Sub Network Nodes Selection Rules, some nodes in W are selected as the set of (7). The Cartesian product of the set describes the couplings between the nodes in the set, and the information about the edge is added to the point set. The operation of finding norm of difference set is the construction process of new sub-network. On the basis of the synchronization stability theory of complex networks, the EEG dynamics equation (7) will be the final state of the nodes in the brain network if the proposed model realize synchronization (whether constant or phase synchronization) at a certain time point.

#### Proof and Optimization of CDBNSs

Proof and Optimization of CDBNSs Model For the brain network equation of the state equation (7), a vibrational equation is obtained by linearizing its synchronization solution at *x*(*t*) = *S*(*t*), let

(8) $$\begin{array}{l} \eta_{i}=x_{i}\left(t\right)-s\left(t\right),\;i=1,2,\ldots ,N \end{array}$$

and substitute (8) into (7) to get the variation equations

(9) $$\begin{array}{l} {\dot{\eta }}_{i}=Df\left(x\right)\eta_{i}-\overset{m}{\underset{i=1}{\sum }}\overset{n}{\underset{j=1}{\sum }}cl_{ij}\underset{\left\{W_{i},M_{i},G_{i}\right\}}{min}C\frac{1}{2t}{\left\|S_{i}-U_{i}V_{i}\right\|}_{F}^{2} \end{array}$$

where ${\dot{\eta }}_{i}$ is the variation in the state of the i-th node, *f* (*x*) is the dynamic equation of a single node system in a brain network; *Df* (*x*) is Jacobian matrix after the *f* (*x*) is linearized (which is the same for all nodes in the synchronous state). *L* is the Laplacian matrix of the brain network, ${\left\|S_{i}-U_{i}V_{i}\right\|}_{F}^{2}$ also denotes $\Gamma \left(x_{i}\right)={\left\|S_{i}-U_{i}V_{i}\right\|}_{F}^{2}$, then (9) can be rewritten as

(10) $$\begin{array}{l} {\dot{\eta }}_{i}=Df\left(x\right)\eta_{i}-\overset{m}{\underset{i=1}{\sum }}\overset{n}{\underset{j=1}{\sum }}cl_{ij}\underset{\left\{S_{i},U_{i},V_{i}\right\}}{\min}C\Gamma \left(x_{i}\right) \end{array}$$

where *xi* is state vector of the i-th node, which denotes the oscillation strength of the nodes in the brain network, and the first derivative denotes the change rate of the oscillation strength. The matrix $c={\left(c_{ij}\right)}_{n\times n}\in R^{n\times n}$is the coupling strength of the network (7), namely, the Laplacian matrix. (*l*_*ij*_)_*M*×*N*_ ∈ *R* denotes the matrix element of the coupling matrix. If there is a connection between nodes *i* and *j*, then *l*_*ij*_ = 1, otherwise *l*_*ij*_ = 0. The diagonal of the matrix *l* element is defined as $l_{i}=\sum_{i=1}^{m}\sum_{j=1}^{n}l_{ij}$.

Considering the randomness during the node selection, the RAN process finally adds all the nodes to the set, the maximum between ness centrality and the average distance change greatly due to the influence of the node selection order between the intermediate networks. Therefore, we do extreme processing for the node sets S,U,V of each network using the block coordinate descent (BCD) algorithm (28), which avoids changes in network synchronization stability resulting from the change in the maximum betweenness centrality and the average distance. The specific process is as follows: Establish the relationship between the whole brain node *Si* and the decomposition matrix*U*_*i*_*; V*_*i*_, where λ_1_; λ_2_; λ_3_ are the decomposition coefficients:

(11) $$\begin{array}{l} \left\{S_{\left(i\right)}^{+}\right\}=\text{arg }\underset{S_{i}}{min}\overset{m}{\underset{i=1}{\sum }}\frac{1}{2t_{i}}{\left\|S_{i}-U^{+}V_{i}^{+}\right\|}_{F}^{2} \end{array}$$

(12) $$\begin{array}{l} \left\{V_{\left(i\right)}^{+}\right\}=\text{arg }\underset{V_{i}}{min}\overset{m}{\underset{i=1}{\sum }}\frac{1}{2t_{i}}{\left\|S_{i}^{-}-U^{+}V_{i}^{+}\right\|}_{iF}^{2}\\ \;+\lambda_{1}\overset{n}{\underset{i=1}{\sum }}\frac{1}{2t_{i}}{\left\|V_{i}\right\|}_{F}^{2}+\lambda_{2}\overset{n}{\underset{i=1}{\sum }}\frac{1}{2t_{i}}{\left\|V_{i}R_{i}\right\|}_{F}^{2} \end{array}$$

(13) $$\begin{array}{l} \left\{U_{\left(i\right)}^{+}\right\}=\text{arg }\underset{U\geq 0}{min}\overset{n}{\underset{i=1}{\sum }}\frac{1}{2t_{i}}{\left\|S_{i}^{-}-U^{-}V_{i}^{-}\right\|}_{F}^{2}+\lambda_{3}\|U\| \end{array}$$

During the generation of the nth networks, $\left\|S_{i}^{-}-U^{+}V_{i}^{+}\right\|$ is a local minimum at the linear solution *i* = *t*, (11), (12), and (13) are combined by using the BCD algorithm, and let $P_{\Omega_{i}}$be each node matrix set, then we can get

(14) $$\begin{array}{l} S_{\left(i\right)}^{+}=P_{\Omega_{i}}\left(U^{+}V_{i}^{+}\right)+P_{\Omega_{i}}\left(X_{i}\right) \end{array}$$

where *X*_*i*_ is the brain network that substitutes into (7), *U*_*i*_*, V*_*i*_ is the decomposition matrix. Substituting (14) into (10), we get:

(15) $$\begin{array}{l} {\dot{\eta }}_{i}=Df\left(x\right)\eta_{i}+\overset{n}{\underset{j=1}{\sum }}ca_{ij}\left[P_{\Omega_{i}}\left(U^{+}V_{i}^{+}\right)+P_{\Omega_{i}}\left(X_{i}\right)\right]\\ \;=Df\left(x\right)\eta_{i}+\overset{n}{\underset{j=1}{\sum }}ca_{ij}P_{\Omega_{i}}\left(U^{+}V_{i}^{+}\right)+\overset{n}{\underset{j=1}{\sum }}ca_{ij}P_{\Omega_{i}}\left(X_{i}\right)\\ \;=D\Theta \left(x,x_{i}\right)+\overset{n}{\underset{j=1}{\sum }}ca_{ij}P_{\Omega_{i}}\left(U^{+}V_{i}^{+}\right) \end{array}$$

$D\Theta \left(x,x_{i}\right)=Df\left(x\right)+\overset{n}{\underset{j=1}{\sum }}ca_{ij}P_{\Omega_{i}}\left(X_{i}\right),P_{\Omega_{i}}\left(U^{+}V_{i}^{+}\right)\in \left\{\eta_{1},\eta_{2},\ldots ,\eta_{n}\right\}$, which is the generated brain network according to the above model, when all the brain nodes are taken into account, *Xi* denotes the whole brain network, and $P_{\Omega_{i}}\left(X_{i}\right)=P_{\Omega }\left(S\right)$. Comparing (5) with (15), we can see that the CDBNS model eliminates the existence of complex variables, so that the general MSF in dynamics can be applied in the brain network in the form of matrix decomposition and also the state parameters. When the system stabilizes, the state parameter changes from one to two in the generation process.

### Synchronization Stability Discriminant Construction of CDBNS Model

Equation (15) is the final pattern of the steady-state equation for the brain network. To determine the stability of the model, we needed to construct its Lyapunov function to judge the positive and negative properties of the exponential and, thus, judge the stability of the network. A Lyapunov function of the brain network dynamics system was constructed by using the MSF and the stability of this system can be judged by the sign of the exponents for this function. Considering that the complex network system (15) is a continuous-time dissipative coupling network, if the synchronous steady-state model synchronizes at *s*(*t*), *J*(*t*) is the Jacobian matrix at *f* (*x*(*t*)) = *s*(*t*) and applies *J*(*t*) to the brain network, using the following Lyapunov function:

(16) $$\begin{array}{l} V_{i}\left(t\right)=x_{i}^{T}\left(t\right)S_{i}x_{i}\left(t\right)+\int_{t-\tau }^{\tau }x_{i}^{t}\left(s\right)U_{i}x_{i}\left(s\right)ds\\ \;+\int_{-\tau }^{0}\int_{\beta }^{0}{ẋ}_{i}^{T}\left(t+\alpha \right)V_{i}{ẋ}_{i}\left(t+\alpha \right)d\alpha d\beta \end{array}$$

where *S*_*i*_ > 0, *U*_*i*_ > 0, *V*_*i*_ > 0 is the nodes matrix of the brain network, *V*_*i*_(*t*) is the Lyapulov function, *x*_*i*_(*t*) is the state of node *i* at time *t*, $x_{i}^{T}\left(t\right)$is the transpose matrix of *x*_*i*_(*t*), τ is a short time interval, *alpha* and *beta* are the duration of the experiment. Diagnose *V*_*i*_(*t*), we can get:

(17) $$\begin{array}{l} {\dot{V}}_{i}\left(t\right)={ẋ}_{i}^{T}\left(t\right)S_{i}x_{i}\left(t\right)+x_{i}^{T}\left(t\right)S_{i}{ẋ}_{i}\left(t\right)+{ẋ}_{i}^{T}\left(t\right)U_{i}x_{i}\left(t\right)\\ \;-{ẋ}_{i}^{T}\left(t-\tau \right)U_{i}x_{i}\left(t-\tau \right)+\tau {ẋ}_{i}^{T}\left(t\right)V_{i}x_{i}\left(t\right)\\ \;-\int_{t-\tau }^{\tau }x_{i}^{t}\left(\alpha \right)V_{i}x_{i}\left(\alpha \right)d\alpha =\frac{1}{\tau }\int_{t-\tau }^{t}\sum \left(t,\alpha \right)d\alpha \end{array}$$

(18) $$\begin{array}{l} \sum \left(t,\alpha \right)={ẋ}_{i}^{T}\left(t\right)S_{i}x_{i}\left(t\right)+x_{i}^{T}\left(t\right)S_{i}{ẋ}_{i}\left(t\right)+{ẋ}_{i}^{T}\left(t\right)U_{i}x_{i}\left(t\right)\\ \;-{ẋ}_{i}^{T}\left(t-\tau \right)U_{i}x_{i}\left(t-\tau \right)+\tau {ẋ}_{i}^{T}\left(t\right)V_{i}\dot{x_{i}}\left(t\right)\\ \;-\tau {ẋ}_{i}^{T}\left(\alpha \right)V_{i}\dot{x_{i}}\left(\alpha \right) \end{array}$$

According to the Newton-Leibniz formula, we can get:

(19) $$\begin{array}{l} x_{k}\left(\alpha \right)-x_{k}\left(t-\tau \right)=\int_{t-\tau }^{t}{ẋ}_{k}\left(\alpha \right)d\alpha \end{array}$$

Since the function (19) satisfied any constructive matrices *S*_*i*_, *U*_*i*_, *V*_*i*_, we get the final Lyapunov function

(20) $$\begin{array}{l} d_{b_{ij}}\left(i\right)=\frac{1}{b_{ij}\left(b_{ij}-1\right)}\underset{ij}{\sum }c^{2}+\int_{0}^{t}\frac{1}{2\left(1-t\right)}dt \end{array}$$

*P*_Ω_*i*__ is a dynamic subnet constructed according to the RAN method, t represents the duration of the signal in the brain network, *b*_*ij*_represents the number of nodes connected by the same node, and the number on the diagonal represents the opposite number of rows or columns in the matrix. *d*_*b*_*ij*__(*i*) corresponds to the point that reflects the stability of the characteristic mode (negative denotes stability, positive denotes instability). If all characteristic modes corresponding to the decomposition coefficient are stable, the homogeneous manifold of the network is considered to be asymptotically stable at this coupling strength.

## Numerical Experiment

### Experimental Simulation and Result Analysis on Alcoholism Addiction Data Set

Alcoholism addiction data (https://archive.ics.uci.edu/ml/datasets/EEG+Database) come from the data set of the University of California, Irvine, used for machine learning. This original data set recorded the multi-channel EEG time sequences of two groups, which included 77 alcoholics and 45 participants of the control group. In this experiment, the data of 61-channe scalp EEG in two sets of volunteers was collected, and the electrode position was based on the extended international 10–20 system, the sampling set at 256 Hz, using the modified working-memory task experimental paradigm. Every participant did 120 experiment tasks, and every task was randomly chosen from three different experimental conditions, including single stimulation, double matching stimulation and double nonmatching stimulation. Single stimulation (S1) means presenting only one picture, double matching stimulation (S2 Match) refers to presenting two identical pictures within a short time, and double no-matching stimulation (S2 No Match) means presenting two different pictures within a short time. Moreover, no answer was needed for single stimulation, but the participants under double stimulations were required to judge whether the two figures were the same and press buttons to reply. Data from 20 alcohol abuse patients and 20 normal subjects were selected for the simulation.

Based on previous work by our research group (29, 30), the signals were decomposed by wavelet packet decomposition. The signals were divided into five frequency bands of δ(1–4)Hz, θ(4–8)Hz, α(8–13)Hz, β(13–30)Hz, and γ(30–45)Hz, and the two significant bands of α and θ were selected for analysis. Each subject's brain function network was generated within a connection density of 12–40% (2% step) (31, 33). EEG dynamic equations *f* (*xi*) proposed by Liley (28) is:

(21) $$\begin{array}{l} \left(x\right)=\{\begin{array}{c} \tau \frac{dh_{e}}{dt}=\left(h_{er}-h_{e}\right)+\frac{h_{eeq}-h_{e}}{\left|h_{eeq}-h_{e}\right|}I_{ee}+\frac{h_{ieq}-h_{e}}{\left|h_{ieq}-h_{e}\right|}I_{ie}\\ \tau \frac{dh_{i}}{dt}=\left(h_{ir}-h_{i}\right)+\frac{h_{eeq}-h_{i}}{\left|h_{eeq}-h_{i}\right|}I_{ee}+\frac{h_{ie}-h_{i}}{\left|h_{ieq}-h_{i}\right|}I_{ie}\\ \frac{d^{2}I_{ee}}{d^{t^{2}}}+2\alpha \frac{dI_{ee}}{dt}+\alpha^{2}I_{ee}=Aae\left\{N_{ee}S_{e}\left(h_{e}\right)+p_{ee}\right\}\\ \frac{d^{2}I_{ie}}{d^{t^{2}}}+2\alpha \frac{dI_{ie}}{dt}+\alpha^{2}I_{ie}=Bbe\left\{N_{ie}S_{i}\left(h_{i}\right)+p_{ie}\right\}\\ \frac{d^{2}I_{ei}}{d^{t^{2}}}+2\alpha \frac{dI_{ei}}{dt}+\alpha^{2}I_{ei}=Aae\left\{N_{ei}S_{e}\left(h_{e}\right)+p_{ei}\right\}\\ \frac{d^{2}I_{ii}}{d^{t^{2}}}+2\alpha \frac{dI_{ii}}{dt}+\alpha^{2}I_{ii}=Bbe\left\{N_{ii}S_{i}\left(h_{i}\right)+p_{ii}\right\} \end{array} \end{array}$$

**Figure 2:**
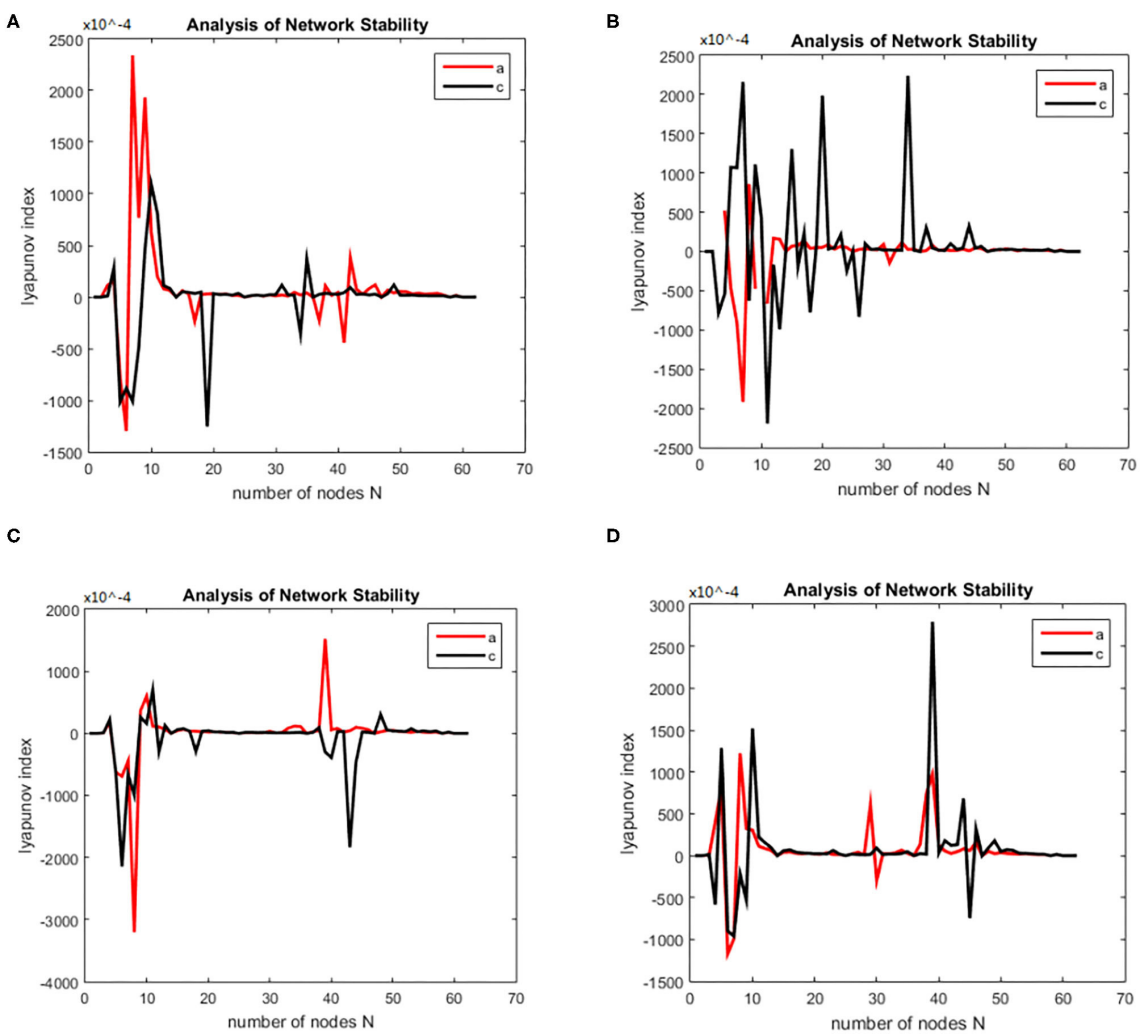
Bain network synchronization experimental results for different subjects in the alcoholic data set under the same connection density (40%). **(A)** Alcoholic subject number is co2a0000405, normal subject number is co2c0000357, **(B)** Alcoholic subject number is co2a0000417, normal subject number is co2c0000367, **(C)** Alcoholic subject number is co2a0000418, normal subject number is co2c0000370, **(D)** Alcoholic subject number is co2a0000451, normal subject number is co2c0000393.

**Figure 3:**
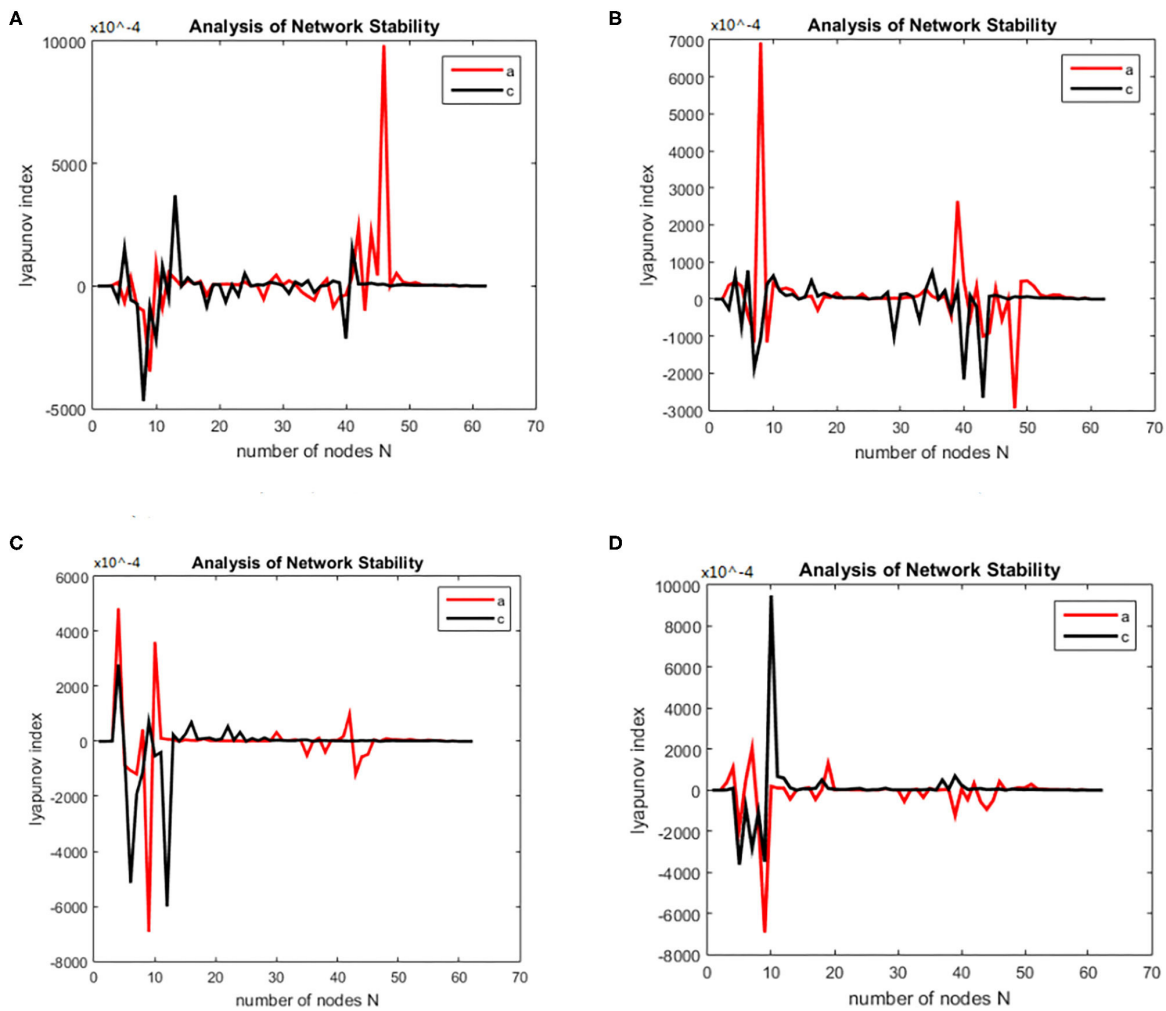
Bain network synchronization experimental results for the same subject in the alcoholic data set under different connection densities. **(A)** Connection density 30%, **(B)** connection density 32%, **(C)** connection density 34%, **(D)** connection density 36%.

Then we solved (21) and transformed the equation into a first order nonlinear system of equations with ten coupling equations with the ten variables *a* = 0.49 ms^−1^*, b* = 0.52 ms^−1^*, A* = 0.81 mV*, B* = 4.85 mV*, e*_*max*_ = *i*_*max*_ = 0.5 ms^−1^*, h*_*eep*_ = 45 mV*, H*_*iep*_ = –90 mV*, h*_*ei*_= *h*_*ir*_ = –70 mV*, N*_*ee*_ = *N*_*ei*_ = 3,034*, N*_*ie*_ = *N*_*ii*_= 536*, p*_*ie*_ = *p*_*ii*_ = 0*, s*_*e*_ = *s*_*i*_= 5 mV, θ_*e*_ = θ_*i*_= –50 mV, τ_*e*_ = 9 ms, τ_*i*_ = 39 ms*, Df* (*x*)was calculated by taking the above initial values into the dynamic equation, and the brain network node set *S* was orthogonally decomposed to obtain the local brain network node set *U, V*. The Lyapunov functions of (7) were, respectively simulated in two groups; the first group was the change in the Lyapunov exponent of the alcoholic patients and the normal subjects in the alpha frequency band, and the second group was the change in Lyapunov exponent of the alcoholic patients and the normal subjects in the theta band. The synchronization tests of the brain network were performed on different subjects in the alcoholics and normal subjects' groups, and the simulation results are shown in Figure 2. The synchronization tests of brain network were performed on different connection densities in alcoholics and normal subjects, and the simulation results are shown in Figure 3.

### Simulation Results Analysis

**Figure 4:**
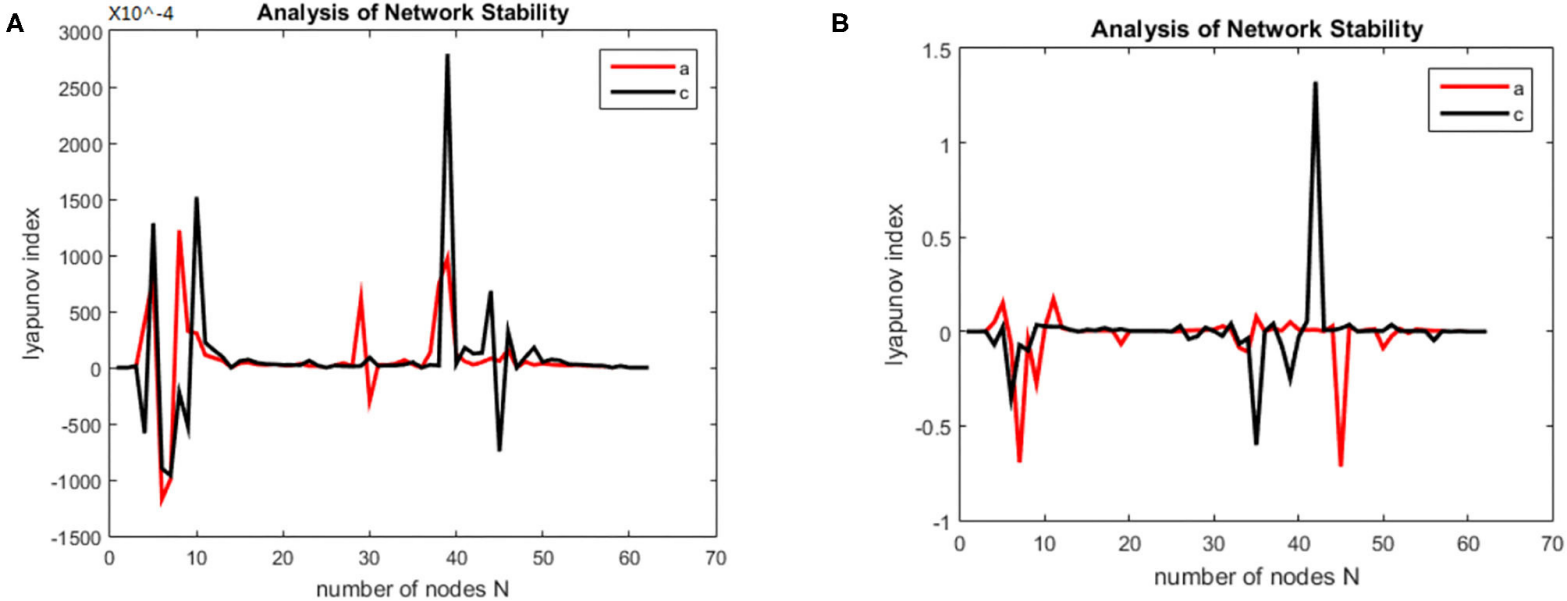
Changes in the Lyapunov exponents in different frequency bands. **(A)** Change of Lyapunov exponent in alpha band. **(B)** Change of Lyapunov exponent in theta band.

Each time the simulation experiment takes a node from the network node set S and adds it to *P*Ω, according to the EEG alcohol public data set description, each experiment time was 3.906 × 80 ms. We selected the EEG data of the alcohol abuse patient number co2a0000364 and normal subject number 339; next we constructed a brain function network for analysis, and took the connection density at 40%. The experimental simulation results are shown in Figures 4A,B. As can be seen in the figure, the Lyapunov exponent of the entire network became negative as the nodes were gradually added to the model, that is, the model reached a synchronized state. A conclusion can be drawn from the above analysis that the CDBNS model can be applied to different connection densities and different frequency bands in the alcoholism data set. If we take Figure 4A as an example to make a detailed analysis of the results, the abscissa indicates the number of added nodes in the brain functional sub-network generated using the random Apollo method. For each added node in the network, a Lyapunov exponent value could be obtained by using (20).

Each node and its number in the alcoholism data set S = {FP1 chan0# FP2 chan1# F7 chan2# F8 chan3# AF1 chan4# AF2 chane5# FZ chan6# F4 chan7# F3 chan8# FC6 chan9# FC5 chan10# FC2 chan11# FC1 chan12# T8 chan13# T7 chan14# CZ chan15# C3 chan16# C4 chan17# CP5 chan18# CP6 chan19# CP1 chan20# CP2 chan21# P3 chan22# P4 chan23# PZ chan24# P8 chan25# P7 chan26# PO2 chan27#PO1 chan28# O2 chan29# O1 chan30# X chan31# AF7 chan32# AF8 chan33# F5 chan34# F6 chan35# FT7 chan36# FT8 chan37# FPZ chan38# FC4 chan39# FC3 chan40# C6 chan41# C5 chan42# F2 chan43# F1 chan44# TP8 chan45# TP7 chan46# AFZ chan47# CP3 chan48# CP4 chan49# P5 chan50# P6 chan51# C1 chan52# C2 chan53# PO7 chan54#PO8 chan55# FCZ chan56# POZ chan57# OZ chan58# P2 chan59# P1 chan60# CPZ chan61# and chan62# Y chan63#}. In Figure 4A the node sequence set U1 added in the normal (i.e., c-line) sub-network generation process = {PZ chan24# CP2 chan21#P4 chan23# POZ chan57# chan42# F8 chan3# PO2 chan27# CP3 chan48# chan50# O2 chan29# T7 chan14# OZ chan58# CP4 chan49# P7 chan26# P3 chan22# C2 chan53# FT7 chan36# P8 chan25#P6 chan51# TP7 chan46# P2 chan59# CP1 chan20# PO1 chan28# TP8 chan45# FC5 chan10# FP2 chan1# T8 chan13# F6 chan35#FCZ chan56# C1 chan52# F7 chan2# F5 chan34# F1 chan44# FC2 chan11# FC6 chan9# C4 chan17# CZ chan15# F4 chan7# FT8 chan37# X chan31# FC1 chan12#AFZ chan47#FP1 chan0#AF8 chan33# F3 chan8# C6 chan41# AF2 chan5# FC3 chan40# CP6 chan19# FC4 chan39# AF7 chan32# PO7 chan54#AF1 chan4# F2 chan43# FPZ chan38# FZ chan6# C3 chan16# PO8 chan55#}. In Figure 4A the node sequence set U2 added in the normal (i.e., a-line) sub-network generation process = {AFZ chan47# F3 chan8# F1 chan44# FCZ chan56#FP2 chan1#FP1 chan0# CZ chan15# FZ chan6# AF1 chan4# FPZ chan 38# X chan31# FC5 chan 10# AF7 chan 32# F5 chan34#FC3 chan40# F7 chan2# AF8 chan33# F6 chan35# FT7 chan36# F8 chan3# AF2 chan5# F2 chan 43# FT8 chan 37# T8 chan13# FC4 chan39# F4 chan 7# FC2 chan11# FC6 chan9# FC1 chan12# T7 chan14# C6 chan41# C5 chan42# C4 chan17# TP8 chan45# TP7 chan46# P6 chan51# P8 chan25# P7 chan26# CP2 chan21#OZ chan58# P4 chan 23# O1 chan 30# PO1 chan28# P3 chan22# CP3 chan48# CP6 chan19# P5 chan50# CP4 chan49# CP5 chan18# P2 chan59# PZ chan24# CP1 chan20# POZ chan57# O2 chan29# PO2 chan27# PO8 chan55# C3 chan16# C2 chan53# C1 chan52#}. It can be seen from Figure 4A that the alcohol abuse patients reached the synchronization earlier than the normal subjects; that is, alcohol appears to have damaged the synchronization of the brain network. When the normal brain network joined the 6th, 7th, and 8th nodes (that is, adding the node F8 chan3# PO2 chan27# CP3 chan48#), the Lyapunov exponent was negative, which indicates that the network had reached a short synchronization at this moment. When the normal brain network joined the 10th, 11th, and 12th nodes (that is, the node O2 chan29# T7 chan14# OZ chan58#), the Lyapunov exponent became a positive number from the previous negative number, indicating that the network was unstable; in particular, the Lyapunov exponent reached a positive maximum of 3.3049 when added to the 26th node (FPZ chan 38#), where the network was the least stable. However, the Lyapunov exponent of the alcoholic patient brain network was negative when the nodes 4, 5, and 6 were added to the brain network of the alcoholism patient (that is, the node FCZ chan 56 # FP2 chan1# FP1 chan0# were added), indicating that the network had reached a short time synchronization. When the brain network of alcoholism patients joined the 7th, 8th, and 9th nodes (that is, added the node CZ chan15 # FZ chan6# AF1 chan4#), the Lyapunov exponent became positive from the previous negative number, indicating that the network was unstable in the following add-on nodes. The Lyapunov exponent of the alcoholism patients' network was roughly distributed around 0. Conclusions can be drawn from the above analysis that the CDBNS model could be applied to different connection densities and different frequency bands in the alcoholism data set.

## Difference Analysis of Synchronization Ability

**Figure 5:**
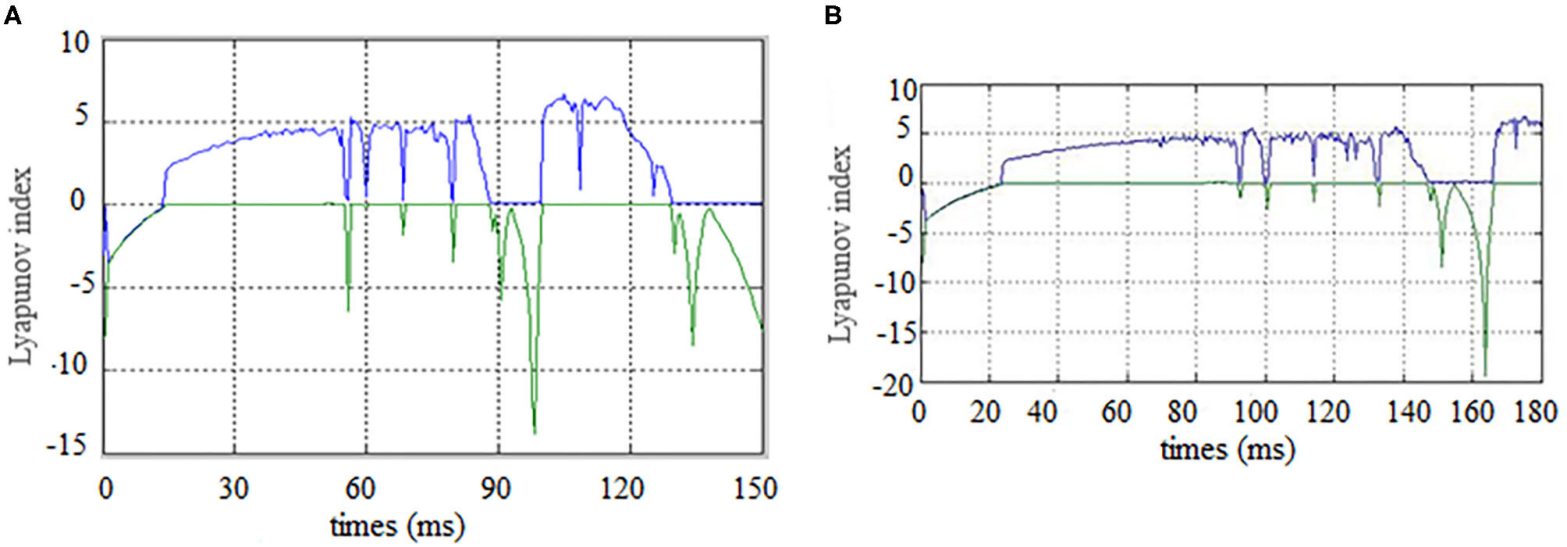
Brain network synchronization status. **(A)** is brain network synchronization status of alcoholic subject co2a0000407; **(B)** is brain network synchronization status of General Subject co2c0000357.

First, the Lyapunov index changes over time (synchronous state of brain networks) were analyzed for alcoholics and healthy subjects, the results are shown in the Figure 5.

As the brain network nodes of the subjects in two groups were gradually added to the model, the max Lyapunov index of the whole network became positive, that is, it reached the synchronous state. For the alcoholism patients in Figure 5A, the max Lyapunov index turned positive from about 15 ms and lasted for about 45 ms until 60 ms. After a short period of instability, the network reached synchronous stable state again, but the duration was shorter. For healthy subjects in Figure 5B, the max Lyapunov index of the entire network becomes positive from about 25 to 150 milliseconds for a duration of about 110 ms.

The alcoholic brain network was synchronized when the sixth node joined the brain network, while the normal subject was synchronized for the first time when the eleventh node joined the brain network. Therefore, the synchronization time is slightly later than that of alcoholics. The normal subject's brain network was also synchronized again and continued for a longer period of time. This is the main difference between the two groups. The longer the time, the less negative maximum Lyapunov index was in the normal subjects compared with the alcoholic patients, which indicates that the synchronization duration of normal human brain network is longer than that of the alcoholic patients on a macro level.

**Figure 6:**
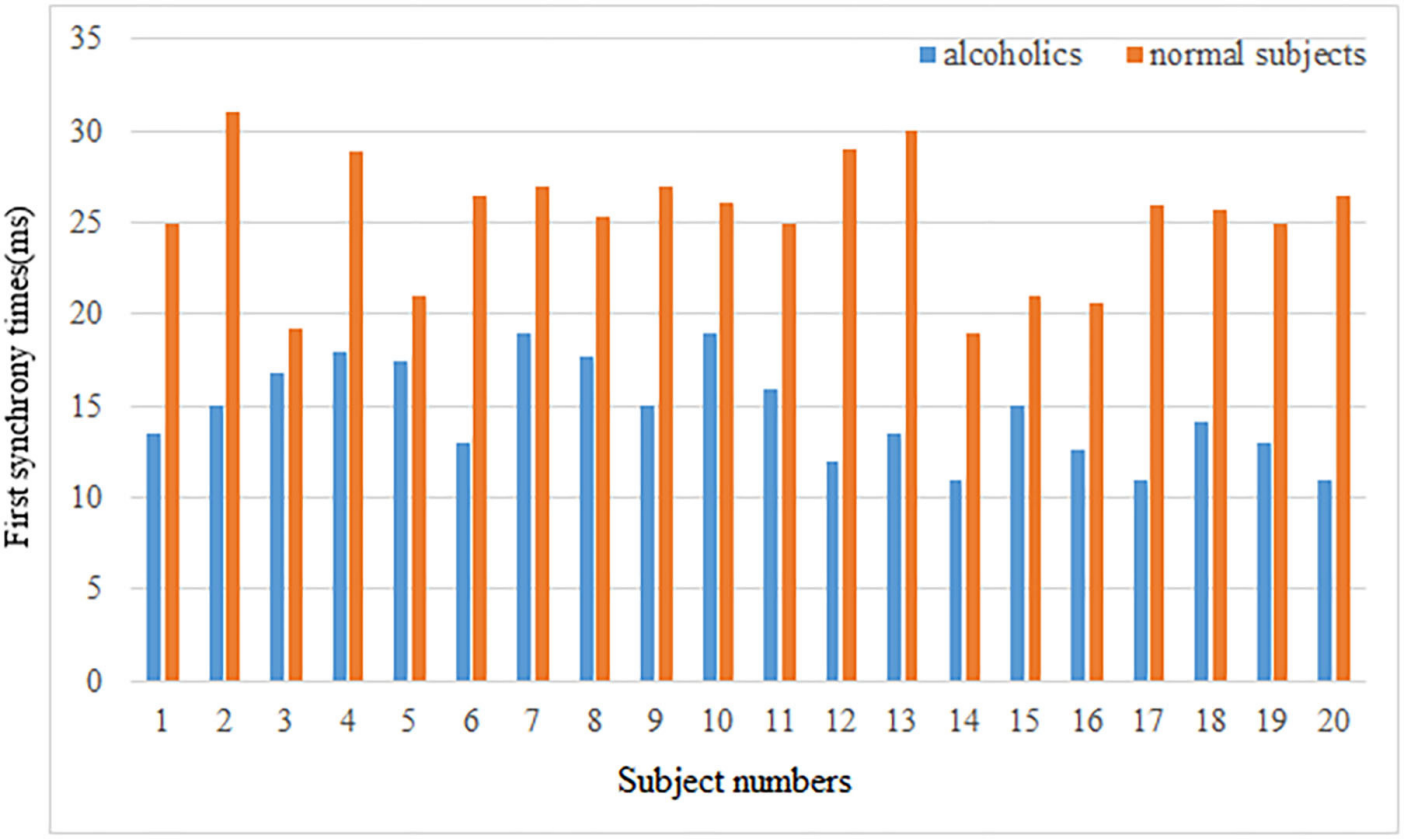
First synchrony times.

Second, the first synchrony time and the first synchrony duration of the brain network Lyapunov index of 20 normal subjects and alcoholics were counted, and the results were shown in the Figure 6.

The mean first synchrony time was 14.67 ± 2.62 ms in the alcoholic group and 25.28 ± 3.46 ms in the healthy control group. The average duration of the first synchrony was 11.66 ± 2.78 ms in the alcoholic group, compared with about 19.92 ± 5.69 ms in the normal group. Although alcoholics achieve synchronous speed more quickly, the duration is shorter. The first synchrony time of normal subjects was later than that of alcoholics, but the duration was longer. Therefore, it can be concluded that the stability of brain network of normal subjects in resting state is better than that of alcoholics.

### Experimental Simulation and Results Analysis on Working Memory Data of Schizophrenia

Experimental Simulation EEG data of working memory of schizophrenia patients collected from Huilongguan Hospital in Beijing. The experimental paradigm used the modified Sternberg's SMST (31) (short-term memory scanning task) paradigm (32), Data from 34 patients with schizophrenia and 34 normal subjects were selected for experiments. Based on previous work by our research group (29–31, 34), none of whom had any record of drug abuse or diagnosis of neuropsychiatric disease in the past 6 months. The age range of the patient group was 20–51 years old, and the average age was (40.1 ± 11.1) years old; the healthy control group age range was 21–58 years old, and the average age was (37.1 ± 13.8) years old. Age, sex, and education level did not differ significantly between the two groups. All the members of both groups had normal vision or corrected visual acuity, had no color disturbance, and were right-handed.

**Figure 7:**
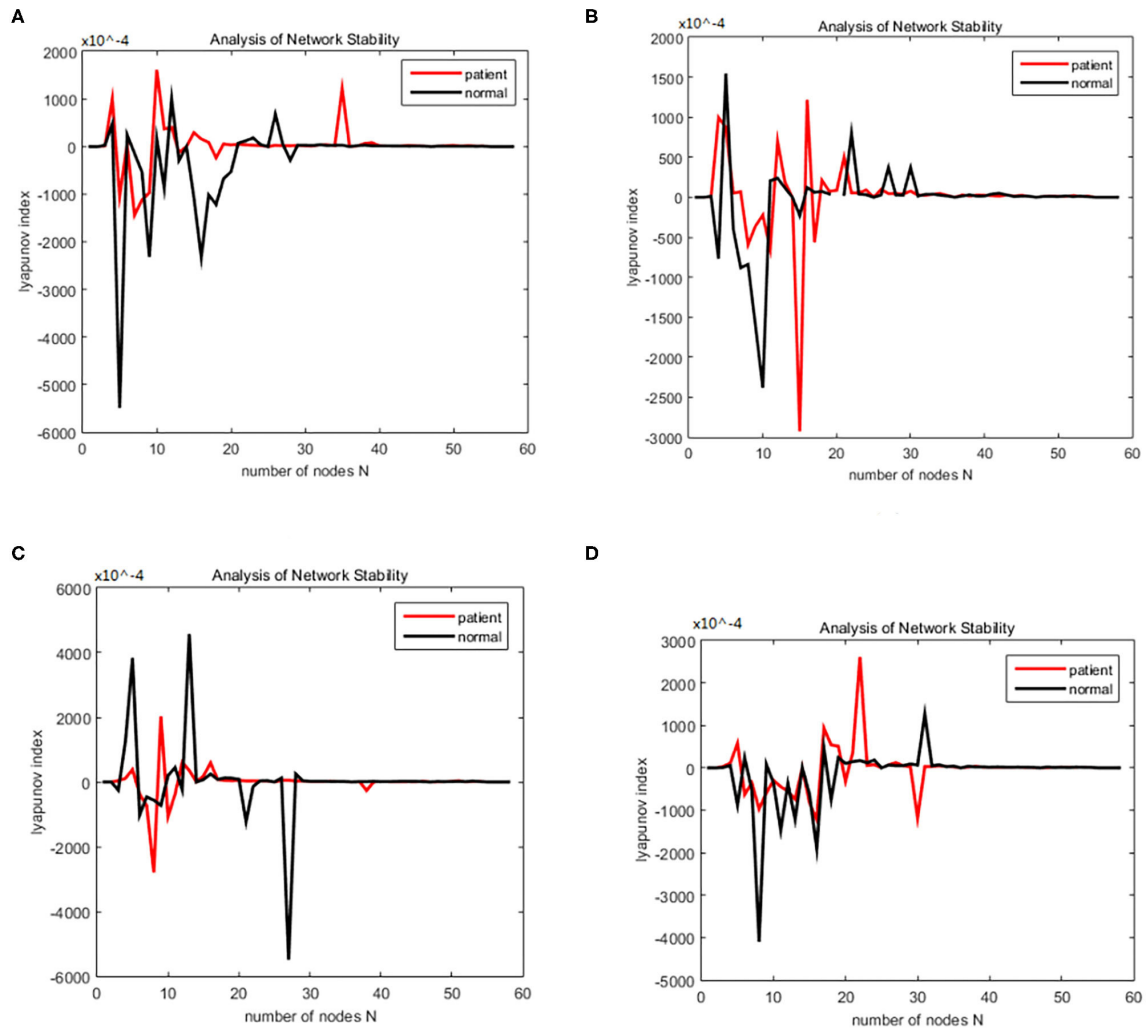
**(A–D)** Brain network synchronization experimental results for different subjects in the schizophrenia data set under the same connection density (40%).

**Figure 8:**
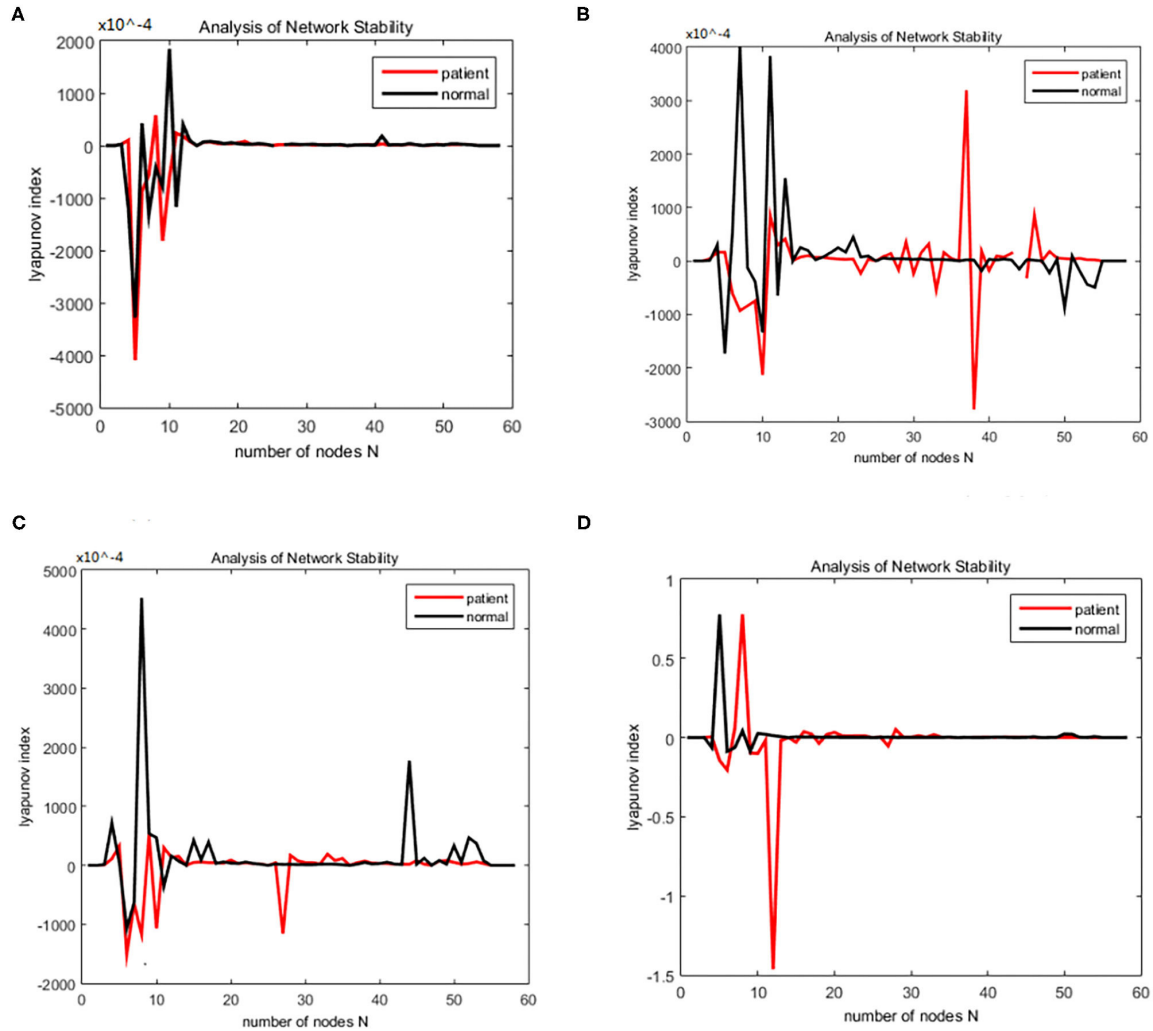
Bain network synchronization experimental results of the same subject in Schizophrenia data set under different connection densities. **(A)** Connection density 30%, **(B)** connection density 32%, **(C)** connection density 34%, **(D)** connection density 36%.

**Figure 9:**
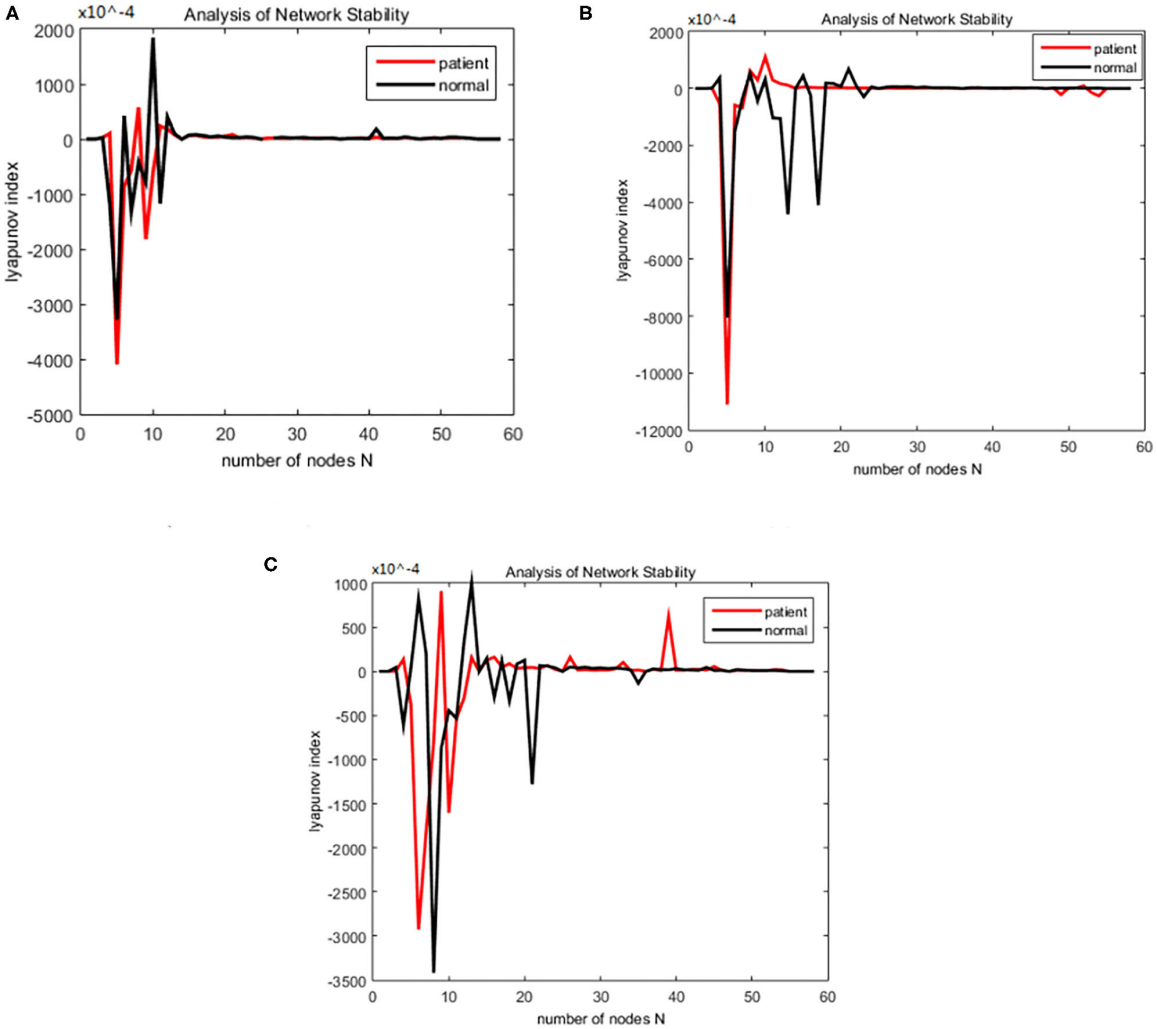
Changes in the Lyapunov exponents during different stages. **(A)** Encoding stage, **(B)** maintenance stage, **(C)** retrieval stage.

The working memory data were divided into three stages: encoding, maintenance, and retrieval. The duration of each stage was 5, 3, and 2.5 s. The EEG data from each stage were divided into alpha and theta frequency bands. The sparseness of 12–40% and the sparseness of 2% were used to generate the brain function network S for each subject. The initialization was the same as for the alcoholism data set and was calculated. The brain nodes set S is orthogonal decomposed into U, V. Using the complex dynamic brain network model synchronization stability discriminant construction in section 2.2 and using the Lyapunov discriminant of (20), six sets of simulations were performed separately using MATLAB. In the alpha frequency band, three groups of experiments were conducted separately, and three groups of experiments were carried out on the theta band separately. The synchronization test of the brain network was performed on different subjects in the schizophrenia and normal subjects, and the simulation results are shown in Figure 7: The synchronization test of the brain network was performed at different connection densities in the schizophrenia patients and normal subjects, and the simulation results are shown in Figure 8: Simulation Results Analysis Taking the simulation on the alpha band as an example, each time the simulation experiment took a node from the network node set S, it was added to *P*Ω. We selected the EEG data of schizophrenia patient number 1098 and normal subject number 1448, constructed the brain function network for analysis, and took the connection density of 40%. The experimental simulation results are shown in Figures 9A–C. The above analysis supports the idea that the CDBNS model can be applied to different connection densities and different frequency bands in a schizophrenia working memory data set. We took the results shown in Figure 9C as an example to do a detailed analysis of the results. The abscissa indicates the number of added nodes in the brain functional sub-network generated using the random Apollo method. For each added node in the network, a Lyapunov exponent value was obtained by using (20). Each node and its number in the schizophrenia working memory data set S= {Ch5 Fp1# Ch6 F7# Ch7 Fp2# Ch8 F3# Ch9 FC3# Ch10 FT7# Ch11 T7# Ch12 F8# Ch13 F4# Ch14 Fz# Ch15 FCz# Ch16 C3# Ch17 TP7# Ch18 FT8# Ch19 FC4# Ch20 Cz# Ch21 CPz# Ch22 CP3# Ch23 P3# Ch24 P7# Ch25 T8# Ch26 TP8# Ch27 C4# Ch28 P8# Ch29 CP4# Ch30 P4# Ch31 O2# Ch32 O1# Ch33 Pz# Ch34 Oz# Ch35 Fpz# Ch36 AF3# Ch37 AF7# Ch38 F5# Ch39 AF8# Ch40 AF4# Ch41 F1# Ch42 FC5# Ch43 F6,Ch44 F2# Ch45 FC1# Ch46 C5# Ch47 FC6# Ch48 FC2# Ch49 C23# Ch50 C1# Ch51 CP1# Ch52 CP5# Ch53 P5# Ch54 C6# Ch55 PO3# Ch56 PO7# Ch57 CP6# Ch58 PO8# Ch59 P6# Ch60 CP2# Ch61 PO4# Ch62 P2# Ch63 POz# Ch64 P1#}. In Figure 9C, the node sequence set U1 added in the normal (ie normal-line) subnetwork generation process = {AF3 Ch36# F1 Ch41# P4 Ch30# F4 Ch13# F3 Ch8# CP6 Ch57# FC5 Ch42# O1 Ch32# AF7 Ch3# Fz Ch14# F5 Ch38# F2 Ch44# FC3 Ch9# FT7 Ch10# Ch29# Fp1 CP4# C1 Ch50# Fp2 Ch7# C23 Ch49# FCz Ch15# Fpz Ch35# PO4 Ch61# C3 Ch16# T7 Ch11# F7 Ch6# Cz Ch20# AF4 Ch40# FC1 Ch45# O2 Ch31# FC2 Ch48# P3 Ch23# P7 Ch24# POz Ch63# PO3 Ch55# FC4 Ch19# T8 Ch25# Oz Ch34# PO8 Ch58# TP8 Ch26# Pz Ch33# C5 Ch46# CP5 Ch52# P1 Ch64# PO7 Ch56# C4 Ch27# F8 Ch12# P2 Ch62# P8 Ch28# CP1 Ch51# CPz Ch21# AF8 Ch39# P6 Ch59# FC6 Ch47# F6 Ch43# P5 Ch53# CP3 Ch22# FT8 Ch18# CP2 Ch60# C6 Ch54# }. It can be seen from Figure 9C that the schizophrenia patients reached synchronization sooner than the normal subjects. When the normal brain network joins the 5th, 6th, 7th, and 8th nodes (that is, adding the node Ch6# F7, Ch4# F1), the Lyapunov exponent was negative, indicating that the network had reached a short synchronization at this moment. When the normal brain network joined the 9th node (that is, adding the node Ch50# C1), the Lyapunov exponent became a positive number from the previous negative number, indicating that the network became unstable in a short time. When joining the 11th and 12th nodes (that is, adding the node Ch44# F2 Ch47# FC6), the Lyapunov exponent became negative again, and the network was again in the synchronized state. However, when the fourth node (i.e., adding node Ch13# F4) was added in the network of schizophrenia patients, the Lyapunov exponents became negative, indicating CP6 Ch42# FC5), the Lyapunov exponent immediately changed from positive to negative, and the network was unstable. When joining the 8th, 9th, and 10th nodes (that is, adding the node Ch32# O1 Ch37# AF7 Ch14# Fz), the Lyapunov exponent value turned positive from negative again, and the network reached a short-term synchronization. In the following add-on nodes, the Lyapunov exponent of the schizophrenia patients and the normal networks were roughly distributed around 0 that the network reached short-term synchronization at this moment. Then, adding the fifth, sixth, and seventh nodes (that is, adding the nodes Ch8# F3 Ch57# CP6 Ch42# FC5), the Lyapunov exponent immediately changed from positive to negative, and the network was unstable. When joining the 8th, 9th, and 10th nodes (that is, adding the node Ch32# O1 Ch37# AF7 Ch14# Fz), the Lyapunov exponent value turned positive from negative again, and the network reached a short-term synchronization. In the following add-on nodes, the Lyapunov exponent of the schizophrenia patients and the normal networks were roughly distributed around 0.

#### Difference Analysis of Synchronization Ability

**Figure 10:**
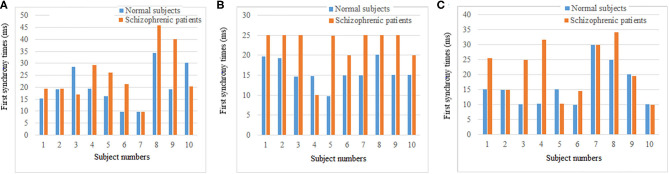
First synchrony time of three stages. **(A)** Encoding stage, **(B)** maintenance stage, **(C)** retrieval stage.

The synchronization stability of the brain networks of the two groups was further determined by the statistics of the first synchronization time. The first synchrony times of 10 subjects in two groups were shown in Figure 10, the average first synchrony times and standard deviation of three stages are showed in Table 1, according to Table 1 and figures, the synchrony time of schizophrenic patients was later than that of healthy subjects, and the standard deviation results showed significant difference in coding stage.

**Table 1:** Mean synchrony time and standard deviation in three stages of two groups.

**Group**	**Stage**	**Mean synchrony time (ms)**	**Standard deviation**
Patient	Encoding	24.83	10.96
	Maintenance	22.53	4.88
	Retrieval	21.54	8.94
Normal	Encoding	20.16	8.33
	Maintenance	15.84	3.09
	Retrieval	16.03	6.98

## Discussion

Lu et al. (9) put forward a series of models related to complex network synchronization, although they have made significant contributions to complex network theory, these type of models often consist of many equations and many parameters and their applicability on large ensembles of elements is highly questionable, which also holds for a bifurcation analysis. Hövel et al. (3) presents a summary of a data-driven computational model of synchronization between distant cortical areas that share a large number of overlapping neighboring (anatomical) connections, in which the coupled oscillatory systems were represented by the Kuramoto phase oscillators. The focus of this modeling approach is to characterize topological properties of functional brain correlation related to synchronization of the regional neural activity. The best agreement between model and experimental data is reached for dynamical states that exhibit a balance of synchrony and variations in synchrony providing the integration of activity between distant brain regions. The limitation of this model is given by its purpose, which does not consider the role of coupling topologies that correspond directly to structural connectivity data. From the perspective of complex network synchronization control, Ferrari et al. (35) presented a model in which cortical areas are represented by networks composed of coupled Rulkov neurons. They improved the stable partial synchronization of the network by adjusting the coupling strength while the intensity of phase synchronization between the cortical areas varies depending on coupling strength. That is how to construct the structural connection matrix has great effect to dynamical patterns in the network. This paper proposes a suitable predictive brain network synchronization model based on EEG signals. It not only proves the validity of the model through formula derivation and theory, but also uses the public alcoholic data set and the real data set collected by the hospital to perform simulation experiments to prove the model's effectiveness, it can finally realize the dynamic observation of synchronization changes in the evolution of brain networks. The current study involved a limitation that should be considered. That is the appropriate threshold range needs to be selected for brain network construction and we will investigate the reason for this behavior in the future.

## Conclusion

Through research on the synchronization stability equations of complex networks, a synchronization steady-state model which is suitable for use in brain networks is put forward, and the theoretical basis and proof are given in this paper. That is to say, because the stability of the state equation of the brain network was transformed into a convex optimization problem, this is a new method using the smooth information from non-smooth functions to study the second-order approximation of convex functions. Thus, we obtained a new method for solving the convex optimization problem. To dynamically observe the synchronization changes during the experiment, we generated a dynamic network using random Apollonian networks (RANs) to add nodes and used the block coordinate descent (BCD) method to do extreme treatments to the dynamic sub-network and to do nonnegative matrix factorization on the brain network nodes set. Finally, the problem of the influence of network synchronization stability brought by the change of the maximum between ness and average distance has been solved. As a result, we can better judge the synchronization stability of the brain network.

Through the analysis of synchronization status in different bands and different connection densities, we were able to clearly see the process of change in the brain network synchronization status with the change in the brain network scale. This is an expected result. We also found that the patients with schizophrenia achieved synchronization sooner than the normal patients. The experimental analysis shows that the establishment of the steady state model can well verify the synchronization of the network and potentially can have a wide range of applications in the study of the pathogenic mechanisms of mental diseases. However, additional studies are needed to discover which nodes in the network contribute the most to the overall stability of synchronization and to explore whether there is a reason why the network achieves a synchronous, stable state due to some special nodes.

## Data Availability Statement

The raw data supporting the conclusions of this article will be made available by the authors, without undue reservation.

## Author Contributions

GY finished the draft. ST, RY, and XC finished data collection. HL and LZ finished the design and revised the manuscript. All authors contributed to the article and approved the submitted version.

## Conflict of Interest

The authors declare that the research was conducted in the absence of any commercial or financial relationships that could be construed as a potential conflict of interest.
